# [2,6-Bis(diphenyl­phosphan­yloxy)phenyl-κ^3^ *P*,*C* ^1^,*P*′]hydroxidonickel(II)

**DOI:** 10.1107/S1600536811014267

**Published:** 2011-06-18

**Authors:** Davit Zargarian, Abderrahmen Salah

**Affiliations:** a Département de Chimie, Université de Montréal, CP 6128, Succ. Centre-ville, Montréal, Québec, Canada H3C 3J7

## Abstract

The mol­ecule of the title complex, [Ni(C_30_H_23_O_2_P_2_)(OH)], adopts a slightly distorted square-planar geometry around Ni^II^ defined by the coordination of the two mutually *trans* P atoms, the C*sp* ^2^ atom of the pincer ligand and the O atom of the hydroxide ligand. The largest distortions from ideal geometry are reflected in the smaller than usual P—Ni—P [163.95 (3)°] and P—Ni—C [82.06 (6)°] angles. The OH ligand does not form intra- or inter­molecular hydrogen bonds.

## Related literature

For general background to pincer complexes and their applications, see: Leis *et al.* (2008 ▶); Dijkstra *et al.* (2001 ▶); Naghipour *et al.* (2007 ▶); van der Boom & Milstein (2003 ▶); Nishiyama (2007 ▶).
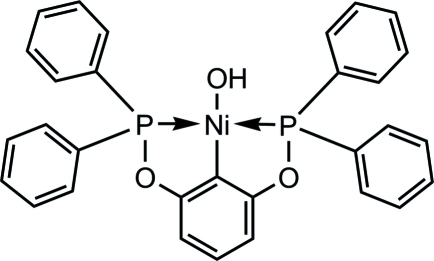


## Experimental

### 

#### Crystal data

[Ni(C_30_H_23_O_2_P_2_)(OH)]
*M* *_r_* = 553.14
Monoclinic, 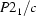

*a* = 15.0626 (7) Å
*b* = 9.8901 (5) Å
*c* = 17.3820 (8) Å
β = 90.150 (2)°
*V* = 2589.4 (2) Å^3^
*Z* = 4
Cu *K*α radiation
μ = 2.49 mm^−1^
*T* = 150 K
0.22 × 0.18 × 0.08 mm

#### Data collection

Bruker SMART 6000 diffractometer
Absorption correction: multi-scan (*SADABS*; Sheldrick, 1996 ▶) *T*_min_ = 0.391, *T*_max_ = 0.819
34083 measured reflections
5038 independent reflections
4673 reflections with *I* > 2σ(*I*)
*R*_int_ = 0.055

#### Refinement

*R*[*F* ^2^ > 2σ(*F* ^2^)] = 0.053
*wR*(*F* ^2^) = 0.143
*S* = 1.07
5038 reflections
327 parameters
H-atom parameters constrained
Δρ_max_ = 0.85 e Å^−3^
Δρ_min_ = −0.91 e Å^−3^

### 

Data collection: *APEX2* (Bruker, 2009 ▶); cell refinement: *SAINT* (Bruker, 2009 ▶); data reduction: *SAINT*; program(s) used to solve structure: *SHELXS97* (Sheldrick, 2008 ▶); program(s) used to refine structure: *SHELXL97* (Sheldrick, 2008 ▶); molecular graphics: *SHELXTL* (Sheldrick, 2008 ▶); software used to prepare material for publication: *UdMX* (Maris, 2004 ▶) and *publCIF* (Westrip, 2010 ▶).

## Supplementary Material

Crystal structure: contains datablock(s) I, global. DOI: 10.1107/S1600536811014267/lh5216sup1.cif

Structure factors: contains datablock(s) I. DOI: 10.1107/S1600536811014267/lh5216Isup2.hkl

Additional supplementary materials:  crystallographic information; 3D view; checkCIF report

## Figures and Tables

**Fig. 1.**
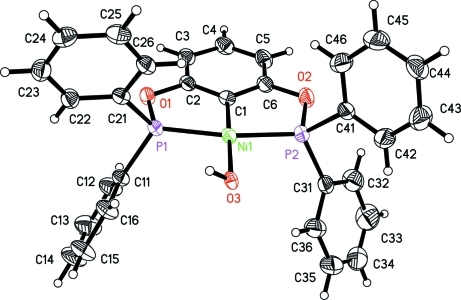
The molecular structure of the title compound, showing 50% probability displacement ellipsoids.

## supplementary crystallographic information

### Comment

Pincer-type complexes have attracted much attention recently due to their promise as functional materials and versatile catalysts (Leis *et al.*, 2008; Dijkstra *et al.*, 2001; Naghipour *et al.*, 2007; van der Boom & Milstein, 2003; Nishiyama, 2007). Herein we report the crystal structure and the synthesis of κ*^P^*,κ*^C^*,κ*^P^*-{*m*-(Ph_2_PO)_2_C_6_H_3_}Ni(OH). The formation of the title complex was unexpected in that the original goal of the synthesis was to prepare the corresponding methyl derivative {*m*-(Ph_2_PO)_2_C_6_H_3_}Ni(CH_3_). Reaction of the trifluoromethanesulfonate precursor with the Grignard reagent MeMgCl did result in the generation of the target methyl derivative, as ascertained by ^31^P and ^1^H NMR spectra of the reaction mixture. It appears, however, that the target methyl complex is not sufficiently stable toward hydrolysis, reacting with residual water during the work-up process to give the observed hydroxo compound. As shown in Fig. 1, the Ni^II^ ion in the title complex exists in the center of a square plane defined by the donor atoms P1 and P2, the C atom of the aromatic moiety of the pincer ligand, and the O atom of the hydroxyl ligand. A slight tetrahedral distortion is evident in the solid state of this complex despite the rigid meridional coordination of the tridentate pincer-type ligand, but such distortions are commonly found in this family of Ni^II^ pincer complexes (van der Boom *et al.*, 2003). Perhaps the most surprising aspect of this structure is the absence of inter- or intramolecular hydrogen-bonding type interactions involving the hydroxy group: the closest O—H distance observed in this structure involved a hydrogen of one of the phenyl substituents, but the distance for this interaction is too long to represent a strong interaction (3.080 Å).

### Experimental

Transfer of MeMgCl (0.12 ml of a 1.8 *M* solution in THF, 0.22 mmol) to a stirred solution of {*m*-(Ph_2_PO)_2_C_6_H_3_}Ni(OSO_2_CF_3_) (50 mg, 0.073 mmol) Br in dry and degassed toluene (1.5 ml) caused an immediate color change from deep-yellow to red. The resulting mixture was stirred under an inert atmosphere of nitrogen for 5 min and was then filtered through cellulose. Evaporation of the solvent gave an orange solid. Single crystals suitable for X-ray diffraction studies were grown by slowly diffusing hexane into a saturated toluene solution. Evaporation of the filtrate gave a red–orange solid, which was crystallized by slow diffusion of hexane into a saturated toluene solution of the crude solid.

### Refinement

All H atoms attached to C atoms were positioned geometrically and refined as riding, with C—H = 0.95 Å, and *U*_iso_(H) = 1.2*U*_eq_(C). The H atom attached to the O atom was positioned geometrically and refined as riding using the AFIX 147 command in SHELXL (Sheldrick, 2008), with O—H = 0.84 Å, and *U*_iso_(H) = 1.5*U*_eq_(C).

### Crystal data

**Table:**

[Ni(C_30_H_23_O_2_P_2_)(OH)]	*F*(000) = 1144
*M**_r_* = 553.14	*D*_x_ = 1.419 Mg m^−^^3^
Monoclinic, *P*2_1_/*c*	Cu *K*α radiation, λ = 1.54178 Å
Hall symbol: -P 2ybc	Cell parameters from 21489 reflections
*a* = 15.0626 (7) Å	θ = 2.9–72.1°
*b* = 9.8901 (5) Å	µ = 2.49 mm^−^^1^
*c* = 17.3820 (8) Å	*T* = 150 K
β = 90.150 (2)°	Block, yellow
*V* = 2589.4 (2) Å^3^	0.22 × 0.18 × 0.08 mm
*Z* = 4	

### Data collection

**Table:**

Bruker SMART 6000 diffractometer	5038 independent reflections
Radiation source: X-ray Sealed Tube	4673 reflections with *I* > 2σ(*I*)
graphite	*R*_int_ = 0.055
Detector resolution: 5.5 pixels mm^-1^	θ_max_ = 72.5°, θ_min_ = 2.9°
ω scans	*h* = −18→18
Absorption correction: multi-scan (*SADABS*; Sheldrick, 1996)	*k* = −12→12
*T*_min_ = 0.391, *T*_max_ = 0.819	*l* = −20→21
34083 measured reflections	

### Refinement

**Table:**

Refinement on *F*^2^	Secondary atom site location: difference Fourier map
Least-squares matrix: full	Hydrogen site location: inferred from neighbouring sites
*R*[*F*^2^ > 2σ(*F*^2^)] = 0.053	H-atom parameters constrained
*wR*(*F*^2^) = 0.143	*w* = 1/[σ^2^(*F*_o_^2^) + (0.1015*P*)^2^ + 1.2898*P*] where *P* = (*F*_o_^2^ + 2*F*_c_^2^)/3
*S* = 1.07	(Δ/σ)_max_ = 0.001
5038 reflections	Δρ_max_ = 0.85 e Å^−^^3^
327 parameters	Δρ_min_ = −0.91 e Å^−^^3^
0 restraints	Extinction correction: *SHELXL97* (Sheldrick, 2008), Fc^*^=kFc[1+0.001xFc^2^λ^3^/sin(2θ)]^-1/4^
Primary atom site location: structure-invariant direct methods	Extinction coefficient: 0.00117 (15)

### Special details

**Table:**

Experimental. X-ray crystallographic data for I were collected from a single-crystal sample, which was mounted on a loop fiber. Data were collected using a Bruker Platform diffractometer, equipped with a Bruker SMART 2K charge-coupled device (CCD) area detector, using the program *SMART* and normal focus sealed-tube source graphite monochromated Cu Kα radiation. The crystal-to-detector distance was 4.908 cm, and the data collection was carried out in 512 × 512 pixel mode, utilizing 4 × 4 pixel binning. The initial unit-cell parameters were determined by a least-squares fit of the angular setting of strong reflections, collected by a 9.0 degree scan in 30 frames over four different parts of the reciprocal space (120 frames total). One complete sphere of data was collected, to better than 0.8 Å resolution. Upon completion of the data collection, the first 101 frames were recollected in order to improve the decay correction analysis.
Geometry. All e.s.d.'s (except the e.s.d. in the dihedral angle between two l.s. planes) are estimated using the full covariance matrix. The cell e.s.d.'s are taken into account individually in the estimation of e.s.d.'s in distances, angles and torsion angles; correlations between e.s.d.'s in cell parameters are only used when they are defined by crystal symmetry. An approximate (isotropic) treatment of cell e.s.d.'s is used for estimating e.s.d.'s involving l.s. planes.
Refinement. Refinement of *F*^2^ against ALL reflections. The weighted *R*-factor *wR* and goodness of fit *S* are based on *F*^2^, conventional *R*-factors *R* are based on *F*, with *F* set to zero for negative *F*^2^. The threshold expression of *F*^2^ > σ(*F*^2^) is used only for calculating *R*-factors(gt) *etc*. and is not relevant to the choice of reflections for refinement. *R*-factors based on *F*^2^ are statistically about twice as large as those based on *F*, and *R*-factors based on ALL data will be even larger.

### Fractional atomic coordinates and isotropic or equivalent isotropic displacement parameters (Å^2^)

**Table:**

	*x*	*y*	*z*	*U*_iso_*/*U*_eq_	
Ni1	0.72577 (2)	0.61980 (3)	0.137780 (17)	0.02246 (9)	
P1	0.61373 (3)	0.53390 (5)	0.19486 (3)	0.02210 (11)	
P2	0.84918 (3)	0.64790 (5)	0.07885 (3)	0.02573 (12)	
O3	0.69443 (7)	0.82471 (12)	0.15693 (7)	0.0184 (2)	
H3	0.6395	0.8362	0.1514	0.028*	
O1	0.62241 (9)	0.36749 (14)	0.18742 (8)	0.0267 (3)	
O2	0.88015 (9)	0.49707 (15)	0.04727 (9)	0.0325 (3)	
C6	0.82528 (13)	0.3945 (2)	0.07568 (11)	0.0275 (4)	
C1	0.75170 (13)	0.43502 (19)	0.11861 (10)	0.0240 (4)	
C25	0.41295 (16)	0.6332 (2)	0.04853 (12)	0.0356 (5)	
H25	0.4079	0.6635	−0.0032	0.043*	
C21	0.50399 (12)	0.56683 (19)	0.15661 (10)	0.0245 (4)	
C4	0.78711 (15)	0.1620 (2)	0.08622 (13)	0.0341 (5)	
H4	0.7991	0.0697	0.0751	0.041*	
C2	0.69701 (12)	0.3306 (2)	0.14473 (10)	0.0246 (4)	
C26	0.49661 (14)	0.6133 (2)	0.08088 (11)	0.0298 (4)	
H26	0.5485	0.6312	0.0516	0.036*	
C41	0.85441 (12)	0.7455 (2)	−0.00910 (11)	0.0276 (4)	
C31	0.94444 (13)	0.6995 (2)	0.13583 (11)	0.0302 (4)	
C12	0.63061 (17)	0.4607 (2)	0.34952 (13)	0.0380 (5)	
H12	0.6536	0.3769	0.3318	0.046*	
C22	0.42718 (13)	0.5421 (2)	0.19899 (12)	0.0291 (4)	
H22	0.4318	0.5105	0.2505	0.035*	
C24	0.33733 (15)	0.6092 (2)	0.09109 (13)	0.0350 (5)	
H24	0.2805	0.6242	0.0689	0.042*	
C46	0.80196 (17)	0.7023 (3)	−0.07062 (14)	0.0436 (6)	
H46	0.7667	0.6232	−0.0655	0.052*	
C3	0.71235 (14)	0.1949 (2)	0.12899 (11)	0.0299 (4)	
H3A	0.6730	0.1269	0.1469	0.036*	
C11	0.60175 (13)	0.5572 (2)	0.29735 (11)	0.0259 (4)	
C23	0.34437 (14)	0.5631 (2)	0.16656 (13)	0.0338 (5)	
H23	0.2923	0.5461	0.1958	0.041*	
C5	0.84496 (14)	0.2614 (2)	0.05923 (12)	0.0333 (4)	
H5	0.8963	0.2381	0.0305	0.040*	
C42	0.90239 (16)	0.8642 (2)	−0.01678 (13)	0.0398 (5)	
H42	0.9375	0.8960	0.0249	0.048*	
C36	0.93527 (16)	0.7970 (3)	0.19298 (14)	0.0449 (6)	
H36	0.8796	0.8400	0.2005	0.054*	
C14	0.5917 (2)	0.6086 (3)	0.45421 (13)	0.0482 (6)	
H14	0.5876	0.6255	0.5079	0.058*	
C16	0.56812 (15)	0.6804 (2)	0.32355 (11)	0.0337 (5)	
H16	0.5485	0.7466	0.2878	0.040*	
C35	1.00701 (19)	0.8316 (4)	0.23897 (15)	0.0560 (7)	
H35	1.0002	0.8982	0.2779	0.067*	
C15	0.56337 (17)	0.7060 (3)	0.40208 (12)	0.0427 (6)	
H15	0.5408	0.7899	0.4200	0.051*	
C44	0.85013 (17)	0.8902 (3)	−0.14670 (13)	0.0426 (6)	
H44	0.8504	0.9382	−0.1940	0.051*	
C34	1.08770 (17)	0.7703 (3)	0.22867 (14)	0.0530 (7)	
H34	1.1365	0.7928	0.2610	0.064*	
C32	1.02697 (16)	0.6403 (3)	0.12424 (16)	0.0464 (6)	
H32	1.0349	0.5758	0.0843	0.056*	
C45	0.80097 (18)	0.7745 (3)	−0.13952 (13)	0.0477 (6)	
H45	0.7661	0.7434	−0.1816	0.057*	
C43	0.89940 (19)	0.9370 (3)	−0.08510 (15)	0.0505 (6)	
H43	0.9314	1.0194	−0.0895	0.061*	
C13	0.6256 (2)	0.4875 (3)	0.42836 (13)	0.0495 (7)	
H13	0.6458	0.4219	0.4643	0.059*	
C33	1.09747 (17)	0.6758 (4)	0.17117 (18)	0.0592 (8)	
H33	1.1536	0.6343	0.1636	0.071*	

### Atomic displacement parameters (Å^2^)

**Table:**

	*U*^11^	*U*^22^	*U*^33^	*U*^12^	*U*^13^	*U*^23^
Ni1	0.02423 (17)	0.02365 (18)	0.01951 (17)	−0.00076 (11)	0.00370 (13)	−0.00133 (11)
P1	0.0237 (2)	0.0242 (2)	0.0184 (2)	0.00024 (16)	0.00292 (17)	0.00010 (15)
P2	0.0247 (2)	0.0286 (2)	0.0239 (2)	−0.00198 (18)	0.00481 (19)	−0.00181 (18)
O3	0.0192 (5)	0.0170 (5)	0.0189 (6)	0.0047 (4)	0.0058 (5)	0.0040 (4)
O1	0.0283 (7)	0.0246 (7)	0.0272 (7)	−0.0003 (5)	0.0069 (5)	0.0005 (5)
O2	0.0325 (7)	0.0310 (7)	0.0341 (8)	−0.0008 (6)	0.0133 (6)	−0.0033 (6)
C6	0.0287 (9)	0.0304 (10)	0.0233 (9)	0.0005 (8)	0.0020 (7)	−0.0007 (7)
C1	0.0265 (9)	0.0252 (9)	0.0204 (8)	0.0008 (7)	−0.0015 (7)	−0.0012 (7)
C25	0.0421 (12)	0.0397 (12)	0.0249 (10)	0.0025 (9)	−0.0075 (9)	0.0005 (8)
C21	0.0282 (9)	0.0251 (9)	0.0203 (8)	0.0002 (7)	−0.0001 (7)	−0.0011 (7)
C4	0.0452 (12)	0.0256 (10)	0.0316 (10)	0.0044 (9)	0.0045 (9)	−0.0034 (8)
C2	0.0262 (9)	0.0290 (9)	0.0186 (8)	0.0012 (7)	0.0007 (7)	−0.0002 (7)
C26	0.0330 (10)	0.0355 (11)	0.0208 (9)	−0.0004 (8)	0.0017 (8)	0.0005 (7)
C41	0.0260 (9)	0.0341 (10)	0.0228 (9)	−0.0006 (8)	0.0035 (7)	−0.0030 (7)
C31	0.0290 (9)	0.0381 (11)	0.0236 (9)	−0.0044 (8)	0.0023 (7)	0.0032 (8)
C12	0.0564 (13)	0.0324 (11)	0.0251 (10)	0.0060 (10)	−0.0026 (9)	0.0019 (8)
C22	0.0280 (9)	0.0334 (10)	0.0258 (10)	−0.0003 (8)	0.0017 (8)	0.0020 (7)
C24	0.0328 (10)	0.0362 (11)	0.0361 (11)	0.0015 (8)	−0.0102 (9)	−0.0028 (8)
C46	0.0505 (13)	0.0464 (13)	0.0338 (11)	−0.0158 (11)	−0.0079 (10)	0.0019 (9)
C3	0.0353 (10)	0.0283 (10)	0.0260 (9)	−0.0010 (8)	0.0023 (8)	0.0009 (7)
C11	0.0294 (9)	0.0304 (9)	0.0180 (8)	−0.0003 (7)	0.0007 (7)	0.0003 (7)
C23	0.0281 (9)	0.0387 (11)	0.0345 (11)	−0.0008 (8)	0.0011 (8)	0.0010 (9)
C5	0.0361 (10)	0.0328 (10)	0.0310 (10)	0.0056 (8)	0.0084 (8)	−0.0033 (8)
C42	0.0433 (12)	0.0470 (13)	0.0292 (11)	−0.0128 (10)	−0.0074 (9)	0.0044 (9)
C36	0.0367 (11)	0.0596 (15)	0.0384 (12)	−0.0042 (11)	0.0006 (9)	−0.0137 (11)
C14	0.0762 (18)	0.0513 (15)	0.0172 (10)	−0.0019 (12)	0.0030 (11)	−0.0007 (9)
C16	0.0450 (11)	0.0358 (11)	0.0203 (9)	0.0075 (9)	0.0010 (8)	−0.0001 (8)
C35	0.0523 (14)	0.0798 (19)	0.0359 (13)	−0.0177 (14)	−0.0031 (11)	−0.0148 (13)
C15	0.0604 (14)	0.0449 (13)	0.0229 (10)	0.0089 (11)	0.0053 (10)	−0.0060 (9)
C44	0.0466 (13)	0.0546 (14)	0.0266 (11)	−0.0014 (11)	−0.0002 (10)	0.0090 (9)
C34	0.0424 (12)	0.084 (2)	0.0327 (12)	−0.0168 (13)	−0.0119 (10)	0.0118 (12)
C32	0.0328 (11)	0.0618 (16)	0.0445 (13)	0.0046 (11)	0.0003 (10)	−0.0037 (11)
C45	0.0559 (14)	0.0569 (16)	0.0301 (11)	−0.0076 (12)	−0.0127 (10)	0.0009 (10)
C43	0.0595 (15)	0.0517 (14)	0.0402 (13)	−0.0207 (12)	−0.0083 (11)	0.0135 (11)
C13	0.0806 (18)	0.0448 (13)	0.0231 (11)	0.0010 (13)	−0.0074 (11)	0.0077 (9)
C33	0.0314 (12)	0.085 (2)	0.0617 (17)	0.0043 (13)	−0.0066 (12)	0.0037 (16)

### Geometric parameters (Å, °)

**Table:**

Ni1—C1	1.8984 (19)	C22—C23	1.383 (3)
Ni1—O3	2.1075 (12)	C22—H22	0.9500
Ni1—P1	2.1361 (5)	C24—C23	1.393 (3)
Ni1—P2	2.1428 (6)	C24—H24	0.9500
P1—O1	1.6561 (14)	C46—C45	1.394 (3)
P1—C11	1.8059 (19)	C46—H46	0.9500
P1—C21	1.8095 (19)	C3—H3a	0.9500
P2—O2	1.6570 (15)	C11—C16	1.396 (3)
P2—C41	1.810 (2)	C23—H23	0.9500
P2—C31	1.814 (2)	C5—H5	0.9500
O3—H3	0.8400	C42—C43	1.389 (3)
O1—C2	1.397 (2)	C42—H42	0.9500
O2—C6	1.399 (2)	C36—C35	1.385 (3)
C6—C5	1.379 (3)	C36—H36	0.9500
C6—C1	1.397 (3)	C14—C13	1.378 (4)
C1—C2	1.398 (3)	C14—C15	1.389 (3)
C25—C24	1.380 (3)	C14—H14	0.9500
C25—C26	1.393 (3)	C16—C15	1.390 (3)
C25—H25	0.9500	C16—H16	0.9500
C21—C22	1.395 (3)	C35—C34	1.370 (4)
C21—C26	1.398 (3)	C35—H35	0.9500
C4—C3	1.390 (3)	C15—H15	0.9500
C4—C5	1.396 (3)	C44—C45	1.368 (4)
C4—H4	0.9500	C44—C43	1.381 (4)
C2—C3	1.389 (3)	C44—H44	0.9500
C26—H26	0.9500	C34—C33	1.376 (5)
C41—C42	1.385 (3)	C34—H34	0.9500
C41—C46	1.395 (3)	C32—C33	1.383 (4)
C31—C32	1.389 (3)	C32—H32	0.9500
C31—C36	1.392 (3)	C45—H45	0.9500
C12—C11	1.386 (3)	C43—H43	0.9500
C12—C13	1.398 (3)	C13—H13	0.9500
C12—H12	0.9500	C33—H33	0.9500
			
C1—Ni1—O3	178.55 (7)	C23—C24—H24	120.0
C1—Ni1—P1	82.06 (6)	C45—C46—C41	120.4 (2)
O3—Ni1—P1	97.56 (4)	C45—C46—H46	119.8
C1—Ni1—P2	82.06 (6)	C41—C46—H46	119.8
O3—Ni1—P2	98.38 (4)	C2—C3—C4	117.87 (19)
P1—Ni1—P2	163.95 (3)	C2—C3—H3A	121.1
O1—P1—C11	102.24 (8)	C4—C3—H3A	121.1
O1—P1—C21	102.85 (8)	C12—C11—C16	120.07 (18)
C11—P1—C21	104.24 (9)	C12—C11—P1	121.71 (16)
O1—P1—Ni1	107.24 (5)	C16—C11—P1	118.06 (15)
C11—P1—Ni1	119.25 (7)	C22—C23—C24	120.0 (2)
C21—P1—Ni1	118.67 (6)	C22—C23—H23	120.0
O2—P2—C41	100.79 (9)	C24—C23—H23	120.0
O2—P2—C31	102.18 (9)	C6—C5—C4	117.91 (19)
C41—P2—C31	105.97 (9)	C6—C5—H5	121.0
O2—P2—Ni1	106.67 (5)	C4—C5—H5	121.0
C41—P2—Ni1	120.84 (6)	C41—C42—C43	120.4 (2)
C31—P2—Ni1	117.47 (7)	C41—C42—H42	119.8
Ni1—O3—H3	109.5	C43—C42—H42	119.8
C2—O1—P1	111.40 (12)	C35—C36—C31	120.3 (2)
C6—O2—P2	111.64 (12)	C35—C36—H36	119.9
C5—C6—C1	123.77 (19)	C31—C36—H36	119.9
C5—C6—O2	119.43 (18)	C13—C14—C15	120.2 (2)
C1—C6—O2	116.80 (17)	C13—C14—H14	119.9
C6—C1—C2	115.52 (17)	C15—C14—H14	119.9
C6—C1—Ni1	122.28 (15)	C15—C16—C11	119.9 (2)
C2—C1—Ni1	122.18 (15)	C15—C16—H16	120.0
C24—C25—C26	120.4 (2)	C11—C16—H16	120.0
C24—C25—H25	119.8	C34—C35—C36	120.4 (3)
C26—C25—H25	119.8	C34—C35—H35	119.8
C22—C21—C26	119.39 (18)	C36—C35—H35	119.8
C22—C21—P1	122.17 (14)	C14—C15—C16	119.8 (2)
C26—C21—P1	118.38 (15)	C14—C15—H15	120.1
C3—C4—C5	121.5 (2)	C16—C15—H15	120.1
C3—C4—H4	119.3	C45—C44—C43	120.0 (2)
C5—C4—H4	119.3	C45—C44—H44	120.0
C3—C2—O1	119.44 (17)	C43—C44—H44	120.0
C3—C2—C1	123.46 (19)	C35—C34—C33	119.5 (2)
O1—C2—C1	117.09 (17)	C35—C34—H34	120.3
C25—C26—C21	119.75 (19)	C33—C34—H34	120.3
C25—C26—H26	120.1	C33—C32—C31	119.6 (3)
C21—C26—H26	120.1	C33—C32—H32	120.2
C42—C41—C46	118.7 (2)	C31—C32—H32	120.2
C42—C41—P2	123.86 (16)	C44—C45—C46	120.1 (2)
C46—C41—P2	117.31 (16)	C44—C45—H45	119.9
C32—C31—C36	119.1 (2)	C46—C45—H45	119.9
C32—C31—P2	120.61 (18)	C44—C43—C42	120.3 (2)
C36—C31—P2	120.30 (16)	C44—C43—H43	119.8
C11—C12—C13	119.6 (2)	C42—C43—H43	119.8
C11—C12—H12	120.2	C14—C13—C12	120.4 (2)
C13—C12—H12	120.2	C14—C13—H13	119.8
C23—C22—C21	120.44 (19)	C12—C13—H13	119.8
C23—C22—H22	119.8	C34—C33—C32	121.2 (3)
C21—C22—H22	119.8	C34—C33—H33	119.4
C25—C24—C23	120.0 (2)	C32—C33—H33	119.4
C25—C24—H24	120.0		
			
C1—Ni1—P1—O1	1.08 (8)	Ni1—P2—C41—C42	117.01 (18)
O3—Ni1—P1—O1	179.67 (6)	O2—P2—C41—C46	58.51 (19)
P2—Ni1—P1—O1	−7.35 (11)	C31—P2—C41—C46	164.64 (18)
C1—Ni1—P1—C11	116.43 (9)	Ni1—P2—C41—C46	−58.5 (2)
O3—Ni1—P1—C11	−64.98 (8)	O2—P2—C31—C32	20.6 (2)
P2—Ni1—P1—C11	108.00 (11)	C41—P2—C31—C32	−84.5 (2)
C1—Ni1—P1—C21	−114.70 (9)	Ni1—P2—C31—C32	136.89 (18)
O3—Ni1—P1—C21	63.88 (8)	O2—P2—C31—C36	−158.00 (19)
P2—Ni1—P1—C21	−123.14 (10)	C41—P2—C31—C36	96.9 (2)
C1—Ni1—P2—O2	6.11 (8)	Ni1—P2—C31—C36	−41.7 (2)
O3—Ni1—P2—O2	−172.49 (7)	C26—C21—C22—C23	0.0 (3)
P1—Ni1—P2—O2	14.54 (11)	P1—C21—C22—C23	−177.23 (16)
C1—Ni1—P2—C41	120.10 (10)	C26—C25—C24—C23	−0.9 (3)
O3—Ni1—P2—C41	−58.50 (9)	C42—C41—C46—C45	2.7 (4)
P1—Ni1—P2—C41	128.53 (11)	P2—C41—C46—C45	178.4 (2)
C1—Ni1—P2—C31	−107.71 (10)	O1—C2—C3—C4	179.85 (17)
O3—Ni1—P2—C31	73.69 (9)	C1—C2—C3—C4	1.3 (3)
P1—Ni1—P2—C31	−99.28 (12)	C5—C4—C3—C2	−0.5 (3)
C11—P1—O1—C2	−128.20 (13)	C13—C12—C11—C16	0.1 (4)
C21—P1—O1—C2	123.89 (13)	C13—C12—C11—P1	175.5 (2)
Ni1—P1—O1—C2	−1.99 (13)	O1—P1—C11—C12	22.2 (2)
C41—P2—O2—C6	−134.73 (14)	C21—P1—C11—C12	129.08 (19)
C31—P2—O2—C6	116.14 (14)	Ni1—P1—C11—C12	−95.74 (19)
Ni1—P2—O2—C6	−7.73 (14)	O1—P1—C11—C16	−162.33 (16)
P2—O2—C6—C5	−175.80 (16)	C21—P1—C11—C16	−55.48 (18)
P2—O2—C6—C1	5.3 (2)	Ni1—P1—C11—C16	79.71 (17)
C5—C6—C1—C2	−0.2 (3)	C21—C22—C23—C24	0.1 (3)
O2—C6—C1—C2	178.60 (16)	C25—C24—C23—C22	0.4 (3)
C5—C6—C1—Ni1	−178.51 (16)	C1—C6—C5—C4	1.0 (3)
O2—C6—C1—Ni1	0.3 (2)	O2—C6—C5—C4	−177.81 (19)
P1—Ni1—C1—C6	178.12 (16)	C3—C4—C5—C6	−0.6 (3)
P2—Ni1—C1—C6	−4.23 (15)	C46—C41—C42—C43	−1.2 (4)
P1—Ni1—C1—C2	−0.06 (15)	P2—C41—C42—C43	−176.7 (2)
P2—Ni1—C1—C2	177.59 (16)	C32—C31—C36—C35	−1.8 (4)
O1—P1—C21—C22	76.98 (17)	P2—C31—C36—C35	176.9 (2)
C11—P1—C21—C22	−29.41 (19)	C12—C11—C16—C15	−0.2 (3)
Ni1—P1—C21—C22	−164.91 (14)	P1—C11—C16—C15	−175.71 (18)
O1—P1—C21—C26	−100.31 (16)	C31—C36—C35—C34	0.0 (4)
C11—P1—C21—C26	153.30 (16)	C13—C14—C15—C16	1.0 (4)
Ni1—P1—C21—C26	17.80 (18)	C11—C16—C15—C14	−0.4 (4)
P1—O1—C2—C3	−176.54 (15)	C36—C35—C34—C33	1.3 (5)
P1—O1—C2—C1	2.1 (2)	C36—C31—C32—C33	2.2 (4)
C6—C1—C2—C3	−1.0 (3)	P2—C31—C32—C33	−176.4 (2)
Ni1—C1—C2—C3	177.34 (15)	C43—C44—C45—C46	−1.3 (4)
C6—C1—C2—O1	−179.56 (15)	C41—C46—C45—C44	−1.4 (4)
Ni1—C1—C2—O1	−1.3 (2)	C45—C44—C43—C42	2.7 (4)
C24—C25—C26—C21	1.0 (3)	C41—C42—C43—C44	−1.5 (4)
C22—C21—C26—C25	−0.5 (3)	C15—C14—C13—C12	−1.1 (5)
P1—C21—C26—C25	176.84 (16)	C11—C12—C13—C14	0.5 (4)
O2—P2—C41—C42	−125.99 (19)	C35—C34—C33—C32	−0.9 (5)
C31—P2—C41—C42	−19.9 (2)	C31—C32—C33—C34	−0.9 (5)
